# Substance Abuse among Drivers of Motor Vehicle Collisions

**DOI:** 10.5812/traumamon.4182

**Published:** 2012-05-26

**Authors:** Hojjat Derakhshanfar, Mohamad Kalantari Meibodi, Hamid Kariman, Ali Arhamidolatabadi, Saeed Safari

**Affiliations:** 1 Department of Emergency Medicine, Shahid Beheshti University of Medical Sciences, Tehran, IR Iran; 2 Department of Emergency Medicine, Shiraz University of Medical Sciences, Shiraz, IR Iran

**Keywords:** Substance-Related Disorders, Accidents, Traffic, Trauma Centers, Emergency Service, Hospital

## Abstract

**Background:**

Motor vehicle collisions (MVC) comprise a majority cause of referral to the emergency department (ED). A large proportion of MVC appear to be preventable, if more effective measures against driving after substance abuse can be implemented.

**Objective:**

This study was aimed to investigate the prevalence of substance abuse among drivers of MVC, following road traffic accidents (RTA).

**Materials and Methods:**

This case-control research was conducted from July to October 2007. One hundred MVC drivers admitted in the ED were included as the case group and 110 hospitalized patients, due to nontraumatic causes were used as controls. History of substances abused was obtained and urine samples were tested for opium in both groups. Finally the history and laboratory results of the groups were compared using SPSS 18.

**Results:**

Of the 100 patients in the case group, 39 (39%) were positive for substance abuse (100% males). On the other hand, 49 (44.5%) patients in the control group had positive history or laboratory findings of substance abuse (73.9% male). Opioids were the most common agent abused in both groups. There was no significant difference between two groups regarding the prevalence of substance abuse (P = 0.92).

**Conclusions:**

The prevalence of substance abuse is high among victims of road traffic injury but in equal proportion to the control group. Health education and counseling is needed to reduce substance abuse in the general population although it was not significantly related to the cause of RTA.

## 1. Background

MVCs comprise the major cause of referral to the trauma unit of EDs and comprise one of the leading public health problems throughout the world (1). Substance abuse can increase the possibility of severe accidents by decreasing awareness and slowing reflexes of victims (2). It is more pronounced among fatally injured drivers compared to sober ones (3). A large proportion of MVC appear to be preventable, if more effective measures against driving after intake of alcohol and drugs can be implemented (4). Ethanol still remains the psychoactive substance most frequently identified in the blood of divers killed in RTA (5). Among a sample of 1,118 adult patients admitted to a trauma center in Baltimore, 44% met lifetime criteria for alcohol abuse or dependence and 32% met criteria for current alcohol abuse or dependence when assessed with a structured clinical interview exam (6). Opioids are other large groups of therapeutic drugs which are abused. CNS depression and decreased respiratory rate and tidal volume are well recognized manifestations of opioid abuse. In the absence of standardized screening tools, trauma center staff has been shown to do poorly in identifying such patients when relying on clinical judgment alone (7). Self-report of peritrauma alcohol and other drug use is valid in this context and may even be more reliable than toxicology screens, which are dependent on the amount of time between an injury and arrival to the trauma center (8).

## 2. Objectives

The present study was aimed to investigate the prevalence of substance abuse among the drivers of MVC following RTA.

## 3. Materials and Methods

In this case-control study, 100 drivers of MVC following RTA, which were referred to the ED trauma unit of our Hospital from the beginning of July to the middle of October 2007 were assessed as the case group. On the other hand, 110 hospitalized patients due to non-traumatic causes (infectious disease, chest pain, cerebrovascular accident, psychology, gynecology, et al.) were used as controls. After stabilizing the vital signs, a history for substances abuse was taken from patients and urine samples were tested for presence of opium in both groups. The patients were ensured that the information obtained from them was to remain completely confidential and written informed consent form was obtained before the study started. Finally the history and laboratory results of the groups were compared using SPSS 18. All the patients with unknown history of substance abuse were excluded. There were no age or sex limitations in this study. The urine samples were tested for opium using opium test strips.

## 4. Results

**Figure 1.**
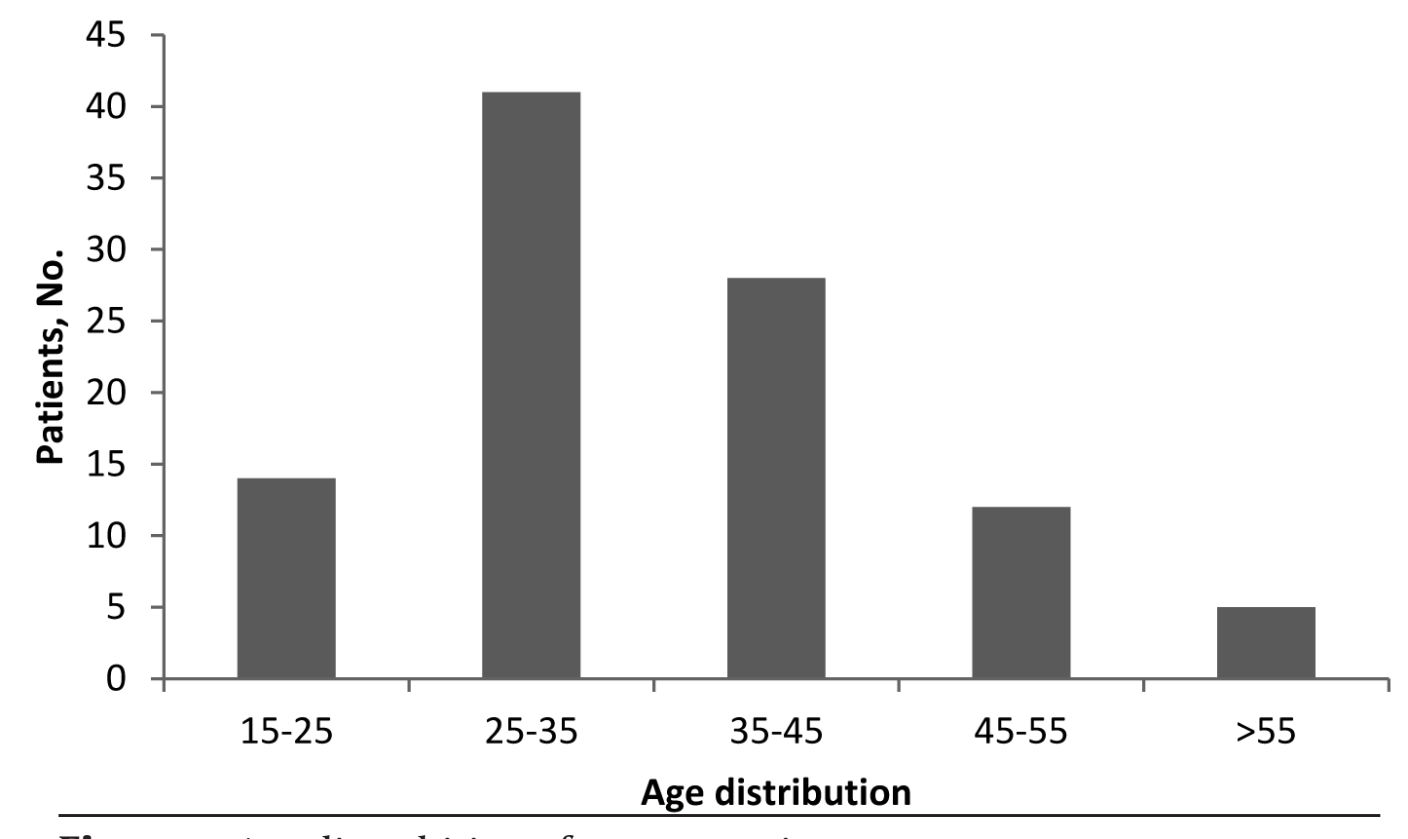
Age distrubition of trauma patients.

**Figure 2.**
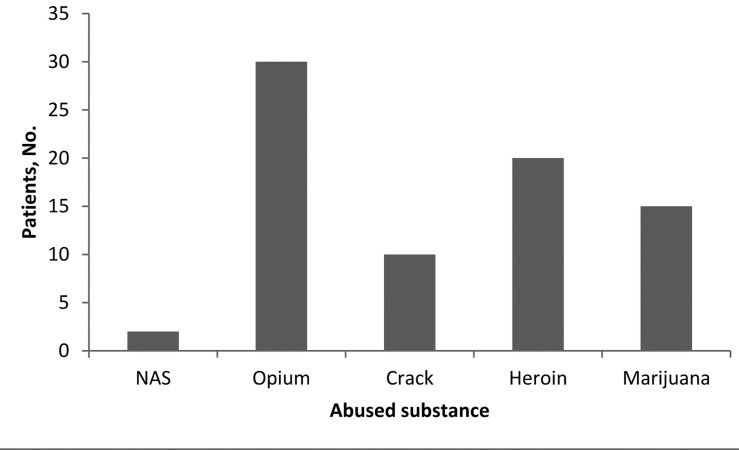
Different types of abused substances among trauma patients (some abuse > 1 type)

Of the total 100 patients comprising the case group, 39 (39%) abused substance based on history or laboratory exam (100% male). The mean age of these patients was 28.6 ± 5.2 years. Figure 1 shows the age distribution among the case group. There was no case of substance abuse among females. Figure 2 shows different type of substances abused by the patients. On the other hand, 49 (44.5%) patients in the control group had positive laboratory findings of substance abuse (73.9% male). The mean age of these patients was 48.9 ± 14.3 years. The mean difference of age between the two groups was significant (P = 0.001). There was no significant difference between two groups regarding the prevalence of substance abuse (P = 0.92). Opioids were common agents abused in the two groups.

## 5. Discussion

The results showed that the prevalence of substance abuse was 39% among the drivers of MVCs who were referred to the ED. This prevalence was 44.5% among control groups. The difference between two groups was not significant. The results of a previous study on opium consumption and the risk of traffic injuries suggested a heightened risk of traffic injuries after opium consumption in regular users (9). A study in Spain showed that abuse of alcohol and cocaine were found in 10.6% and 4% of drivers involved in accidents respectively in 2006; while these proportions were 5.6% and 3.7%, respectively, in 2007 (10). In another study in motorcycle injured Nigerian patients it was shown that alcohol and substance abuse was implicated in 31.2% of them (11). Ricci et al. showed that alcohol and drug presence is common during the weekends (37/43 cases), in contrast to weekdays (6/43 cases) [OR 3.04 (95% CI 1.43; 6.46)]. Alcohol was the most frequently detected abused substance (72%), followed by benzodiazepines, tetrahydrocannabinol and cocaine (12). A large proportion, approximately one-quarter, of trauma patients in Los Angeles County met the criteria for alcohol abuse in the 12 months preceding their injury. Furthermore, 37% reported past 12-month marijuana use and 15% reported use of drugs other than marijuana. After marijuana, cocaine was the most commonly used illegal drug (13). In this study we combined two methods for detection of drug abuse, the first was history of drug abuse in the patients and the second was the urine test. This combined method increased maximum accuracy for the detection of cases of drug abuse and this may be one of the reasons of high detection of drug abuse in this study. The prevalence of substance abuse is high among victims of road traffic injury but in equal proportion to the control group. Health education and counseling is needed to reduce substance abuse in the general population although it was not significantly related to the cause of RTA.
